# Supplementary material for: Deep autoencoder for interpretable tissue-adaptive deconvolution and cell-type-specific gene analysis
Source: Nat Commun. 2022 Nov 8;13:6735. doi: 10.1038/s41467-022-34550-9 (PMC9641692; doi:10.1038/s41467-022-34550-9)
Supplement: Supplementary file 1 — Supplementary Information [file 41467_2022_34550_MOESM1_ESM.pdf]

Supplementary Information for: Deep autoencoder for interpretable tissue-adaptive deconvolution and cell-type-specific gene analysis

Yanshuo Chen[*1,2], Yixuan Wang[*1,3], Yuelong Chen[4,5], Yuqi Cheng[6], Yumeng Wei[1], Yunxiang Li[1], Jiuming Wang[1], Yingying Wei[7], Ting-Fung Chan[†4,5], and Yu Li[†1,8]

[1]Department of Computer Science and Engineering, CUHK, Hong Kong SAR, China [2]School of Life Sciences, Tsinghua University, 100084 Beijing, China
[3]Department of Mathematics, HIT, 264209 Weihai, China
[4]School of Life Sciences, CUHK, Hong Kong SAR, China
[5]State Key Laboratory of Agrobiotechnology, The Chinese University of Hong Kong, Hong Kong SAR, China
[6]Weill Cornell Graduate School of Medical Sciences, Weill Cornell Medicine, New York, NY, 10065, USA
[7]Department of Statistics, The Chinese University of Hong Kong, Hong Kong SAR, China
[8]The CUHK Shenzhen Research Institute, Hi-Tech Park, Nanshan, Shenzhen, 518057, China

Contents

Supplementary Figure 1: Detailed comparison on pseudo-bulk datasets in the "normal" scenario. 2
Supplementary Figure 2: Detailed comparison on pseudo-bulk datasets in the "rare" scenario. 3
Supplementary Figure 3: Detailed comparison on three PBMC real datasets in the "similar" scenario. 3
Supplementary Figure 4: Deconvolution of immune cell subsets. 4
Supplementary Figure 5: Overall performance of all tested methods on five real datasets. 4
Supplementary Figure 6: Detailed comparison between Scaden and TAPE. 5
Supplementary Figure 7: Gene concordance of TAPE and CSx. 5
Supplementary Figure 8: Volcano plots of DEGs calculated from bulk GEPs and inferred GEPs. 6
Supplementary Figure 9: DEG detection would be affected by similar cell types. 6
Supplementary Figure 10: Comprehensive tests for TAPE and CIBERSORTx in four scenarios. 7
Supplementary Figure 11: Data distribution after preprocessing. 8
Supplementary Table 1: Relations between defined cell types and existing cell types in original datasets. 9
Supplementary Table 2: TAPE's performance is affected by variance cut-off. 9
Supplementary Table 3: Hyperparameters tuning for Scaden. 10
Supplementary Table 4: Hyperparameters tuning for RNAsieve. 10
Supplementary Table 5: Hyperparameters tuning for Music. 10
Supplementary Table 6: Hyperparameters tuning for DWLS. 10
Supplementary Table 7: Hyperparameters tuning for CIBERSORTx. 11
Supplementary Table 8: Performance summary of TAPE and SOTA methods. 11
Supplementary Table 9: Performance summary of TAPE and CIBESORTx on the DEG detection task. 12

---

[*]Contribute equally

†Corresponding Author. Email: liyu@cse.cuhk.edu.hk and tf.chan@cuhk.edu.hk

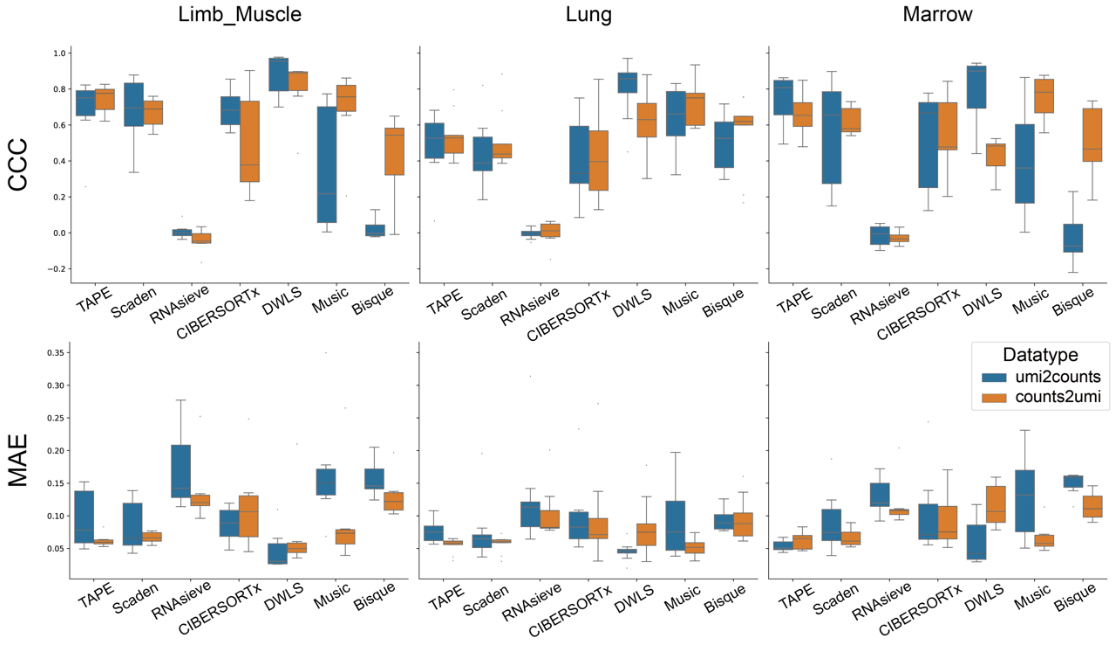

Supplementary Figure 1: Detailed comparison on pseudo-bulk datasets in the "normal" scenario. Each box contains performances of all the cell types in a certain tissue, sample sizes for each box in "Limb_Muscle", "Lung", and "Marrow" are 6, 9, and 7 respectively. In this figure, the boxes represent interquartile range (IQR) while the solid line represents the median. The whiskers extend to points that lie within 1.5 IQRs of the lower and upper quartile, and then observations that fall outside this range are displayed as points independently. Datatype refers to the cross-platform experiments. "umi2counts" means using single-cell profile from UMI-based data as reference to predict pseudo-bulk data constructed from counts-based single-cell profile and vice versa for "counts2umi". We can see many statistical methods suffers from batch effects if the datatype is exchanged. Source data are provided as a Source Data file.

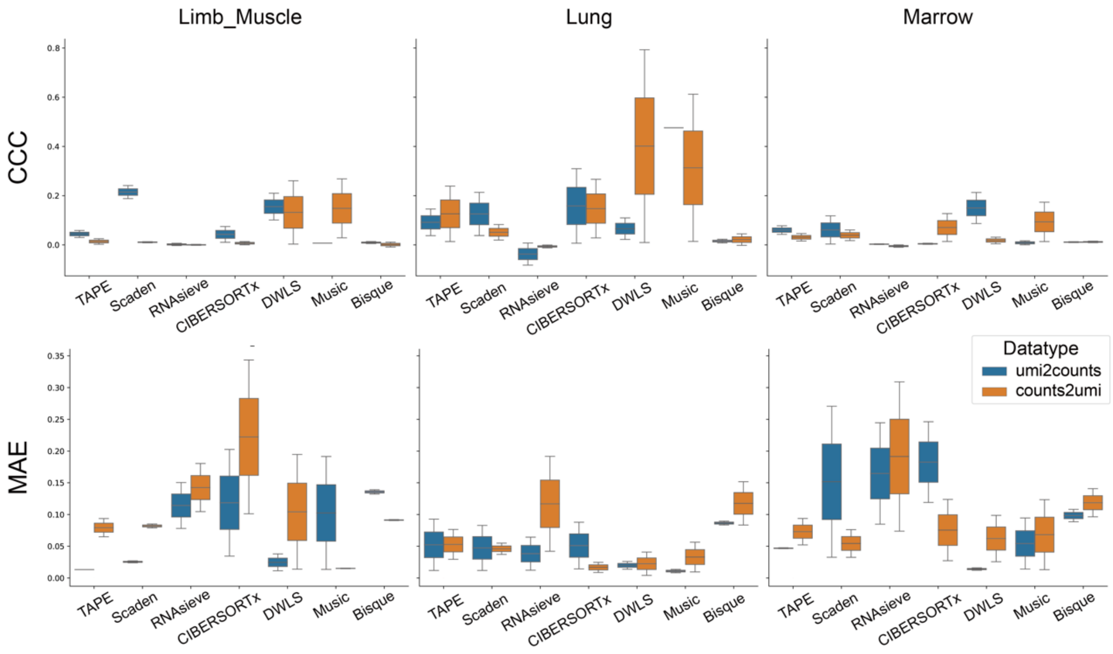

Supplementary Figure 2: Detailed comparison on pseudo-bulk datasets in the "rare" scenario. The figure settings are similar to Supplementary Figure 1 except that only rare cell types are considered in the three simulated datasets. Sample size for each box in each tissue consistently equals to 2. Similar to Supplementary Figure 1, the boxes represent IQR while the solid line represents the median. The whiskers extend to points that lie within 1.5 IQRs of the lower and upper quartile, and then observations that fall outside this range are displayed as points independently. All the methods can not achieve an appealing prediction power for rare cell types. Even though TAPE can achieve a relatively good performance on MAE (for example on umi2counts datatype, TAPE has the smallest average MAE on Limb_Muscle(0.013) and Marrow(0.047)), TAPE has worse performance on CCC in comparison with other methods, which indicates that TAPE needs further improvement in predicting a good correlation for rare cell types. Meanwhile, we have to point out that, for all the methods, a CCC value below 0.3 seems inadequate to show the deconvolution problem being resolved in the 'rare' scenario. Source data are provided as a Source Data file.

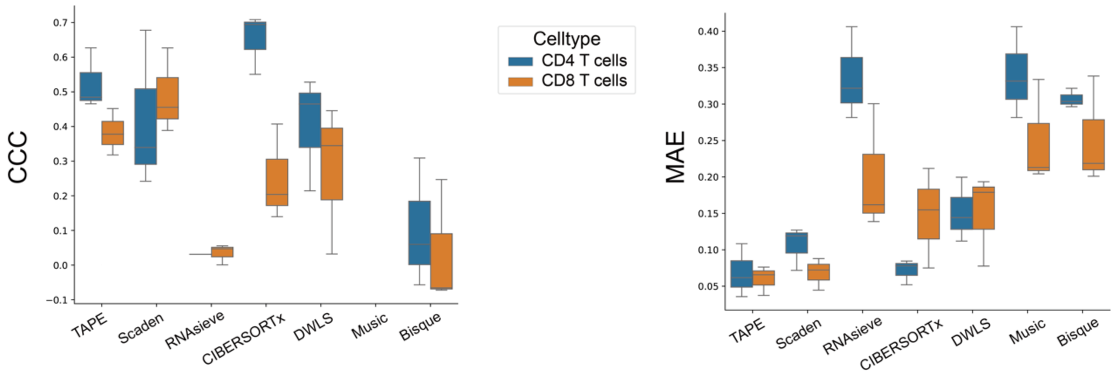

Supplementary Figure 3: Detailed comparison on three PBMC real datasets in the "similar" scenario. In all the three real PBMC datasets we considered, CD4 T cells and CD8 T cells exist in all of them. So we investigated the prediction performance for both of them across three PBMC datasets. Sample size for each box in the figure equals to 5. The boxes represent IQR while the solid line represents the median. The whiskers extend to points that lie within 1.5 IQRs of the lower and upper quartile, and then observations that fall outside this range are displayed as points independently. The results show that TAPE is the best algorithm for distinguishing similar cell types and has stable performance in the two cell types. Source data are provided as a Source Data file.

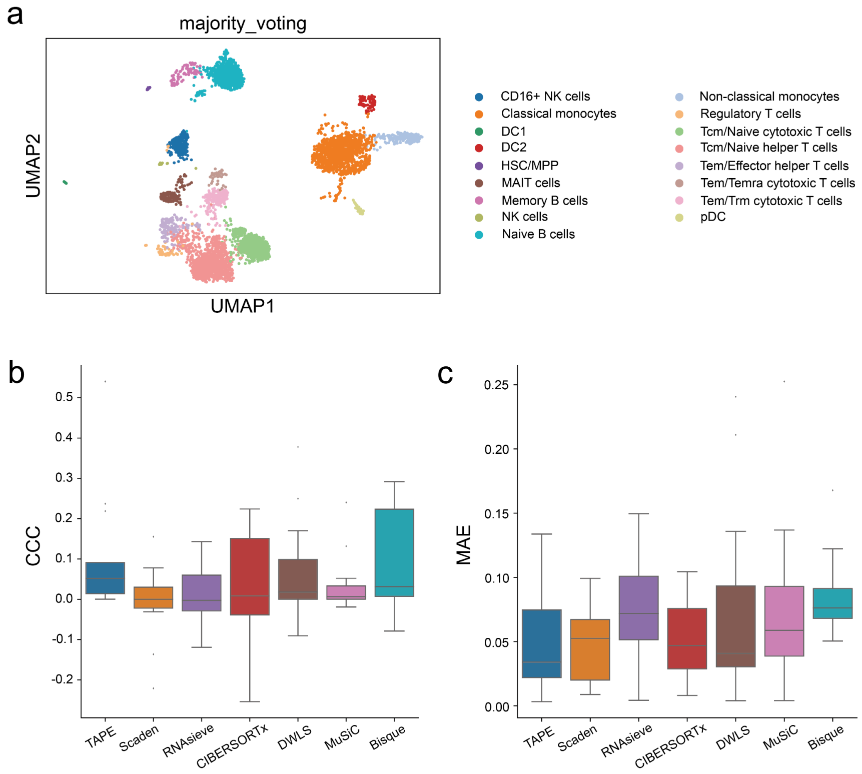

Supplementary Figure 4: Deconvolution of immune cell subsets. a. The annotated cell types of data8k datasets. This is produced from the pipeline of CellTypist [1]. Only cells with a confidence score greater than 0.8 were selected. **b,c.** Deconvolution performance of current methods on Monaco's dataset. Only TAPE predicts positive CCC for all cell types. Source data are provided as a Source Data file.

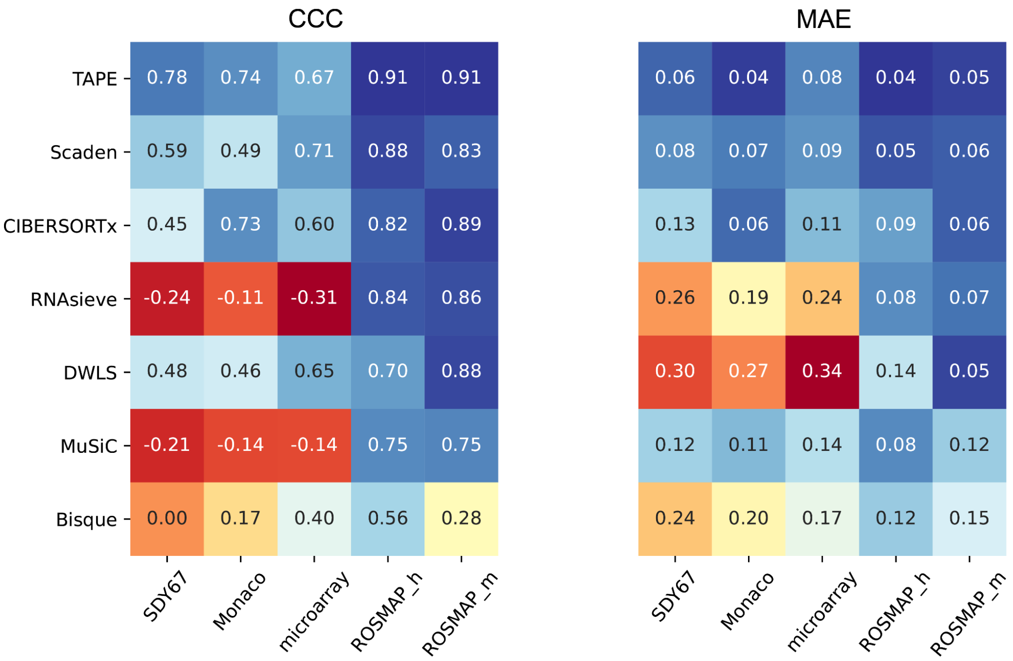

Supplementary Figure 5: Overall performance of all tested methods on five real datasets. The overall performance is calculated by all the data points of a dataset. Source data are provided as a Source Data file.

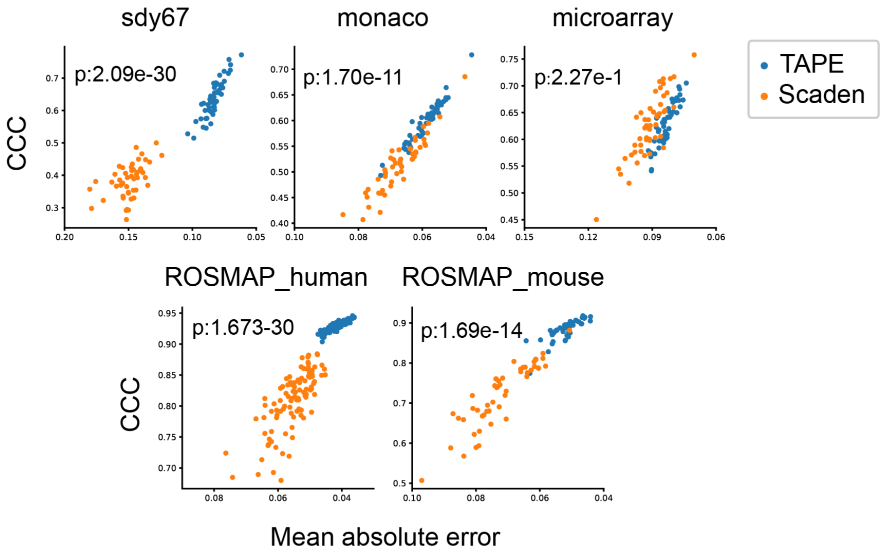

Supplementary Figure 6: Detailed comparison between Scaden and TAPE. A detailed comparison between the performance of Scaden and TAPE on 5 real bulk datasets using 50 different random seeds. The dots on the upper right represent better performance than the dots on the bottom left. Statistical significance is measured by a two-sided t-test which examines the distance from each of the two sets of points to the upper right corner. Source data are provided as a Source Data file.

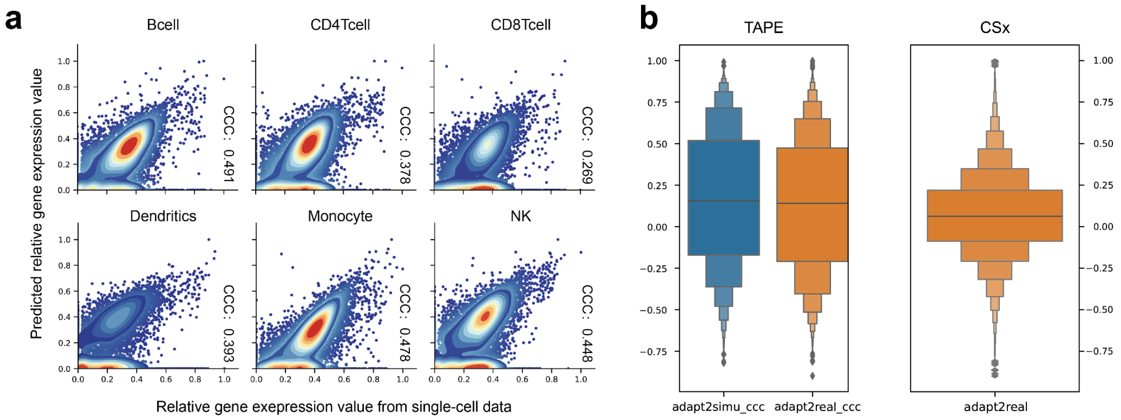

Supplementary Figure 7: Gene concordance of TAPE and CSx. a Concordance between the predicted relative gene expression value in real bulk data and the relative gene expression value in single-cell data of CSx. b Gene level CCC of TAPE and CSx (median CCC of TAPE and CSx in adapt2real scenario are 0.2171 and 0.0627, respectively). Source data are provided as a Source Data file.

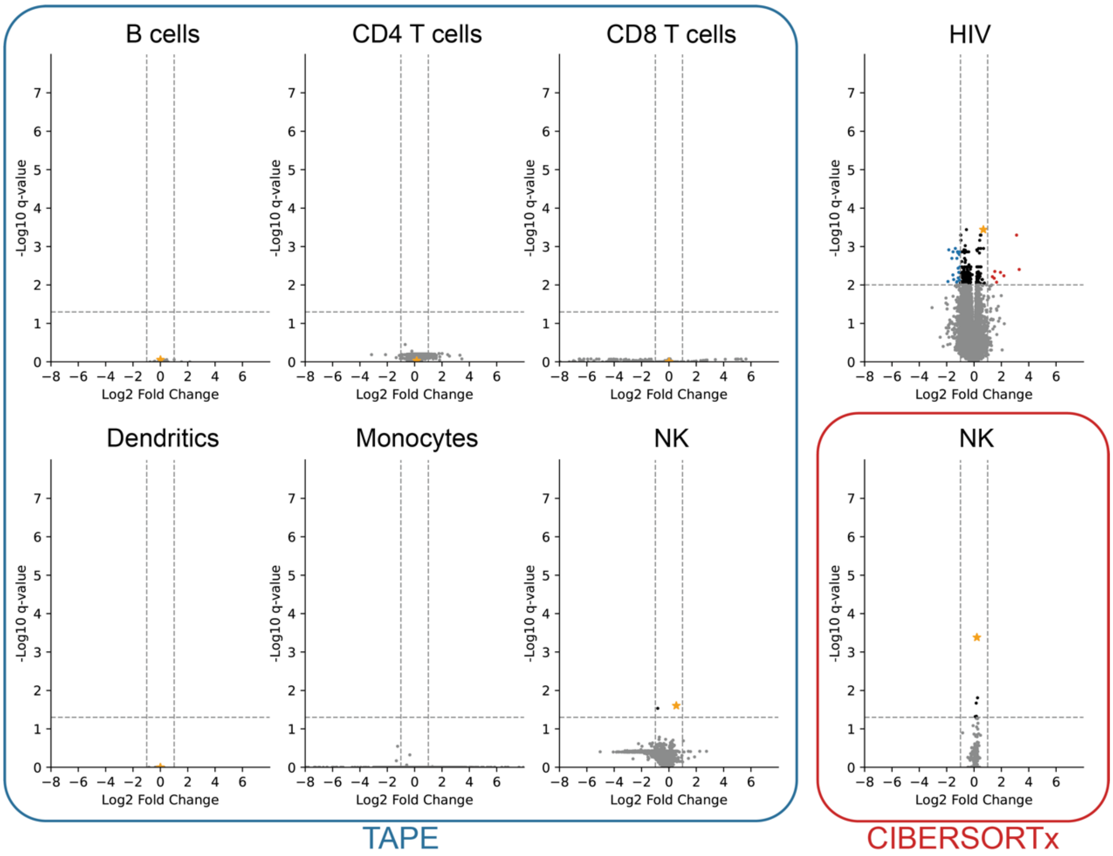

Supplementary Figure 8: Volcano plots of DEGs calculated from bulk GEPs and inferred GEPs. The q-value refers to the p-value adjusted by the false discovery rate. The orange stars refer to the RAB11FIP5 gene. The dash lines refers to the widely used p-value and foldchange criterions (log2(foldchange) > 1, q-value< 0.05 for inferred GEPs and q-value< 0.01 for real bulk GEPs). The DEGs of real bulk are detected using DESeq2 (without filtering out non-related conditions) [2], and the DEGs of inferred GEPs are detected by two-sided t-test. Both CIBERSORTx and TAPE can predict RAB11FIP5 as DEG in NK cells properly, but both methods can not infer a proper foldchange for DEG. Source data are provided as a Source Data file.

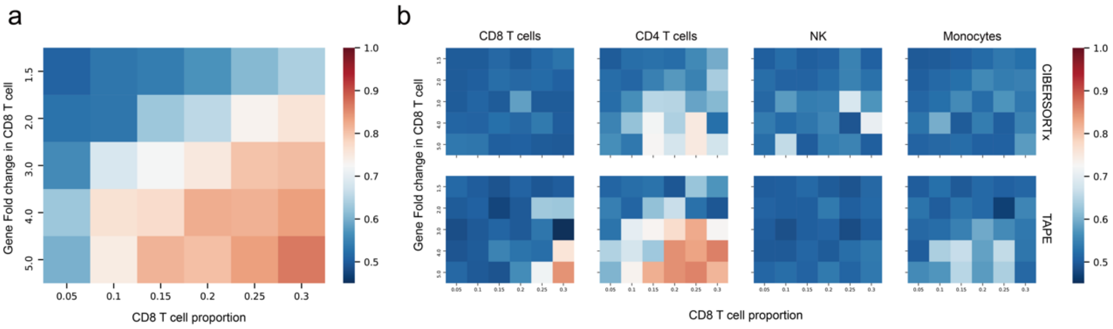

Supplementary Figure 9: DEG detection would be affected by similar cell types (100 randomly selected DEGs with similar cell types). a. Differentially expressed genes detected from simulated bulk RNA-seq data. The color indicates the AUROC value, red means better classification performance. Each row means different up-regulated foldchanges of randomly selected genes in CD8 T cells. Each column means CD8 T cell proportion in simulated bulk data. **b.** Differentially expressed genes detected by CIBERSORTx and TAPE in different cell types. DEGs should only be detected from CD8 T cells. But both methods prefer to think DEGs are from CD4 T cells. Source data are provided as a Source Data file.

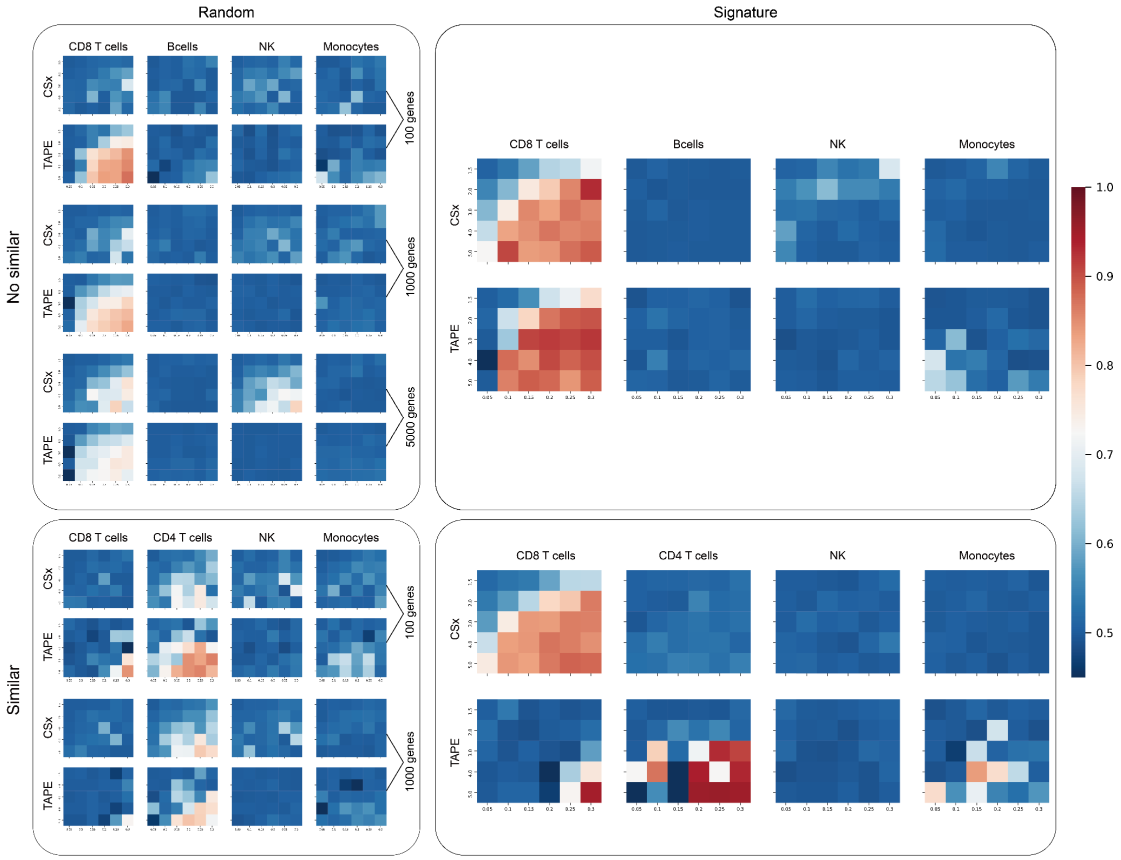

Supplementary Figure 10: Comprehensive tests for TAPE and CIBERSORTx in four scenarios. The upper left scenario uses randomly selected DEGs and it does not contain similar cell types in single-cell profiles. The number of DEGs ranges from 1,00 to 5,000. However, the number of DEGs is usually below 1,000 [3]. The second one is the “signature genes as DEGs without similar cell types” scenario which is located in the upper right. The bottom left area is the “randomly selected DEGs with similar cell types” scenario, and the bottom right one is the ”signature genes as DEGs with similar cell types” scenario. All the tests use AUROC as criteria, and the high AUROC value is expected to only appear in CD8 T cells. In the first scenario, TAPE is better than CIBERSORTx when the number of DEGs is below 5,000 (average AUROC in CD8 T cells for CSx and TAPE are 0.5578 and 0.6538 respectively). In the second scenario, both methods can achieve a good predictive power (average AUROC in CD8 T cells for CSx and TAPE are 0.7639 and 0.7611 respectively). In the third scenario, both methods can not distinguish DEGs from similar cell types well but TAPE’s performance is a little better (average AUROC in CD8 T cells for CSx and TAPE are 0.5146 and 0.5249 respectively). In the last scenario, CIBERSORTx behaves better than TAPE because of the incorporation of the signature matrix (average AUROC in CD8 T cell for CSx and TAPE are 0.7466 and 0.5336 respectively). Source data are provided as a Source Data file.

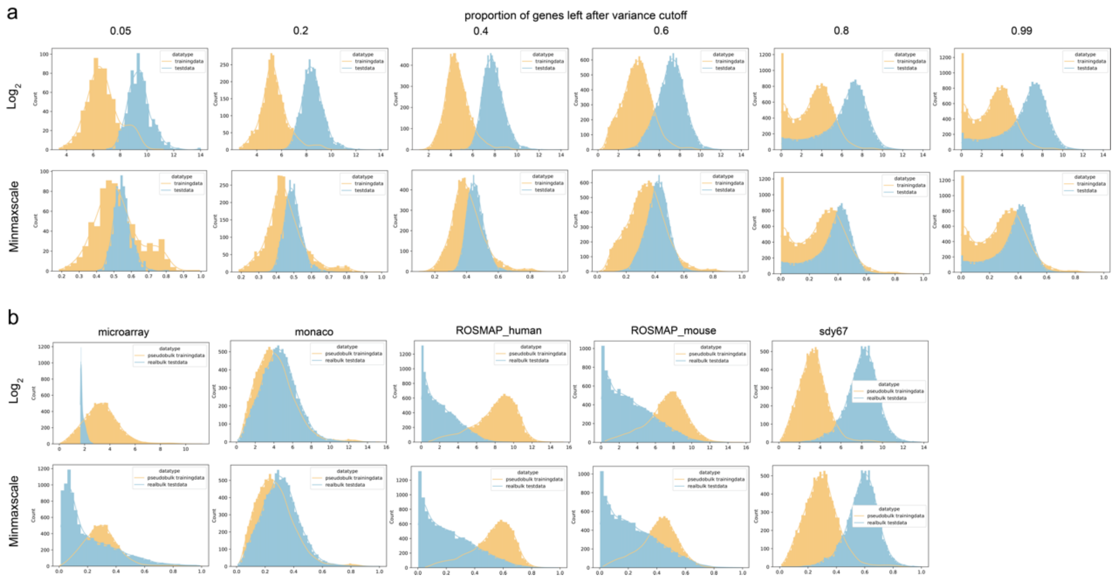

Supplementary Figure 11: Data distribution after preprocessing. In the data preprocessing step, our model needs to filter out low variance genes. a. Data distribution after filtering out different proportions of low variance genes. The test was conducted on the pseudo-bulk test: “Lung umi2counts”. b. Data distribution of all the real bulk tests. Source data are provided as a Source Data file.

Supplementary Table 1: Relations between defined cell types and existing cell types in original datasets. Notice that we merge some cell types to make the categories identical.

Defined cell types	data8k cell types	monaco's cell types
NK	CD16+ NK cells<br>NK cells	NK
Monocytes	Classical monocytes<br>Non-classical monocytes	Monocytes C<br>Monocytes N<br>Monocytes L
mDC	DC1<br>DC2	mDCs
pDC	pDC	pDCs
Naïve B	Naïve B cells	B Naïve<br>B Exhausted
Memory B	Memory B cells	B SM<br>B NSM
MAIT	MAIT cells	MAIT
Naïve CD8 T	Tcm/Naive cytotoxic T cells	T CD8 Naive
Naïve CD4 T	Tcm/Naive helper T cells	T CD4 Naïve
non-Naïve CD4 T	Tem/Effector helper T cells	Tfh<br>Th1<br>Th1/Th17<br>Th17<br>Th2<br>T CD4 TE
non-Naïve CD8 T	Tem/Temra cytotoxic T cells<br>Tem/Trm cytotoxic T cells	T CD8 CM<br>T CD8 EM<br>T CD8 TE
Treg	Regulatory T cells	Tregs
Unknown	HSC/MPP	Progenitors<br>Plasmablasts<br>T gd Vd2<br>Tdg non-Vd2<br>Neutrophils LD<br>Basophiles LD

Supplementary Table 2: TAPE's performance is affected by variance cut-off.

**Fractions of genes left after cutoff**	0.99	0.80	0.60	0.50	0.40	0.20	0.05
**Number of genes left after cutoff**	16599	16529	13200	10718	8076	3489	739
**overall CCC**	0.28	0.28	0.42	0.43	0.59	0.56	0.33
**overall MAE**	0.12	0.12	0.10	0.10	0.07	0.07	0.09

Supplementary Table 3: Hyperparameters tuning for Scaden.

**parameters**	batch size	**128**	64	64	64	64	128	128	128
	learning rate	**1.00E-04**	1.00E-04	1.00E-04	1.00E-05	1.00E-05	1.00E-04	1.00E-05	1.00E-05
	steps	**5000**	2000	5000	2000	5000	2000	2000	5000
**metrics**	CCC_overall	0.49	0.47	0.48	0.56	0.51	0.49	0.51	0.53
	MAE_overall	0.07	0.07	0.08	0.06	0.07	0.07	0.07	0.07
	CCC_average	0.37	0.32	0.32	0.33	0.31	0.31	0.33	0.31
	MAE_average	0.07	0.07	0.08	0.06	0.07	0.07	0.07	0.07

Supplementary Table 4: Hyperparameters tuning for RNAsieve.

**parameters**	trim_percent	**0.02**	0.10	0.05	0.01	0.02	0.10	0.05	0.01	0.02	0.10	0.05	0.01
	gene_thresh	**0.20**	0.20	0.20	0.20	0.10	0.10	0.10	0.10	0.30	0.30	0.30	0.30
**metrics**	CCC_overall	-0.10	-0.08	-0.08	-0.10	-0.06	-0.07	-0.08	-0.06	-0.11	-0.10	-0.11	-0.10
	MAE_overall	0.19	0.17	0.17	0.19	0.18	0.20	0.20	0.18	0.18	0.18	0.18	0.18
	CCC_average	nan	nan	nan	nan	nan	nan	nan	nan	nan	nan	nan	nan
	MAE_average	0.19	0.17	0.17	0.19	0.18	0.20	0.20	0.18	0.18	0.18	0.18	0.18

Supplementary Table 5: Hyperparameters tuning for Music.

**Parameters**	nu	**0.0001**	0.0000	0.0010	0.0100	0.1000	0.0001	0.0000	0.0010	0.0100	0.1000	0.0001	0.0010	0.0100	0.1000	0.0000	0.0001	0.0000	0.0010	0.0100	0.1000
	centered	**FALSE**	FALSE	FALSE	FALSE	FALSE	TRUE	TRUE	TRUE	TRUE	TRUE	FALSE	FALSE	FALSE	FALSE	FALSE	TRUE	TRUE	TRUE	TRUE	TRUE
	normalized	**FALSE**	FALSE	FALSE	FALSE	FALSE	FALSE	FALSE	FALSE	FALSE	FALSE	TRUE	TRUE	TRUE	TRUE	TRUE	TRUE	TRUE	TRUE	TRUE	TRUE
**Metrics**	CCC_overall	-0.0275	0.0249	-0.0262	0.1967	0.2373	0.0986	0.0821	0.1222	0.2324	0.2219	-0.0165	-0.0650	-0.0924	-0.0898	0.0044	-0.0165	0.0280	0.0491	0.0302	-0.0162
	MAE_overall	0.1796	0.1769	0.1683	0.1435	0.1731	0.1885	0.1954	0.1696	0.1496	0.1901	0.1739	0.1741	0.1771	0.1756	0.1712	0.1739	0.1821	0.1739	0.1786	0.1911
	CCC_average	NaN	NaN	NaN	NaN	NaN	NaN	NaN	NaN	NaN	NaN	NaN	NaN	NaN	NaN	NaN	NaN	NaN	NaN	NaN	NaN
	MAE_average	0.1796	0.1769	0.1683	0.1435	0.1731	0.1885	0.1954	0.1696	0.1496	0.1901	0.1739	0.1741	0.1771	0.1756	0.1712	0.1739	0.1821	0.1739	0.1786	0.1911

Supplementary Table 6: Hyperparameters tuning for DWLS.

**parameters**	p-value cutoff	**0.0100**	0.0100	0.0500	0.0500
	diff cutoff	**0.5**	0.5	0.5	0.5
	flavor	**MAST**	Seurat	MAST	Seurat
**Metrics**	CCC_overall	0.4056	0.4358	0.4056	0.1539
	MAE_overall	0.0979	0.1037	0.0979	0.2040
	CCC_average	0.2894	0.1515	0.2894	0.0554
	MAE_average	0.0979	0.1037	0.0979	0.2040

Supplementary Table 7: Hyperparameters tuning for CIBERSORTx.

**parameters**	Kappa	**14.63**	8.98	14.63	8.98	14.63	8.98	14.63	8.98	5	2
	quantile normalization in generating signature matrix	**FALSE**	TRUE	FALSE	TRUE	FALSE	TRUE	FALSE	TRUE	FALSE	FALSE
	S-mode correction	**TRUE**	TRUE	TRUE	TRUE	FALSE	FALSE	FALSE	FALSE	TRUE	TRUE
	quantile normalization in deconvolution	**FALSE**	FALSE	TRUE	TRUE	FALSE	FALSE	TRUE	TRUE	FALSE	FALSE
**Metrics**	CCC_overall	0.6167	0.4955	0.6108	0.5517	0.2260	0.0953	0.2389	0.1061	0.6817	0.5788
	MAE_overall	0.0683	0.0910	0.0659	0.0753	0.0866	0.0996	0.0875	0.0978	0.0603	0.0772
	CCC_average	0.3235	0.2254	0.3189	0.2542	0.3258	0.2504	0.3282	0.2594	0.3477	0.0772
	MAE_average	0.0683	0.0910	0.0659	0.0753	0.0866	0.0996	0.0875	0.0978	0.0603	0.0772

Supplementary Table 8: Performance summary of TAPE and SOTA methods. Here we list the top3 methods in order in different scenarios and datasets. The performance comparison between box plots is evaluated by two-sided t-test. The initial assumption is TAPE's performance is better than other methods. If $p > 0.5$, TAPE's performance is not better than other methods. The order is based on the p-value, a small p-value represents higher performance. In some scenarios with only two data points, we only compute the average value for comparison.

Datatype		Scenario	Dataset	Metrics			
				CCC		MAE	
				Overall	P-value (used for comparing the box plot)	Overall	P-value (used for comparing the box plot)
Real-bulk			sdy67	**TAPE**, Scaden, CSx	DWLS, Scaden, **TAPE**	**TAPE**, Scaden, MuSiC	**TAPE**, Scaden, CSx
			monaco	**TAPE**, CSx, Scaden	**TAPE**, CSx, Scaden	**TAPE**, CSx, Scaden	**TAPE**, CSx, Scaden
			microarray	Scaden, **TAPE**, DWLS	Scaden, CSx, **TAPE**	**TAPE**, Scaden, CSx	**TAPE**, Scaden, CSx
			rosmap_h	**TAPE**, Scaden, RNAsieve	**TAPE**, Scaden, MuSiC	**TAPE**, Scaden, MuSiC	**TAPE**, Scaden, MuSiC
			rosmap_m	**TAPE**, CSx, DWLS	RNAsieve, DWLS, **TAPE**	**TAPE**, DWLS, Scaden	**TAPE**, DWLS, Scaden
Pseudo-bulk	umi2counts	normal	Limb_Muscle	DWLS, CIBERSORTx, Scaden	DWLS, CIBERSORTx, Scaden	DWLS, Scaden, CIBERSORTx	Scaden, DWLS, CIBERSORTx
			Lung	DWLS, Bisque, **TAPE**	DWLS, Bisque, **TAPE**	DWLS, Scaden, **TAPE**	DWLS, Scaden, **TAPE**
			Marrow	DWLS, **TAPE**, Scaden	DWLS, **TAPE**, CIBERSORTx	**TAPE**, DWLS, Scaden	**TAPE**, DWLS, CIBERSORTx
		rare*	Limb_Muscle	not applicable	Scaden, DWLS, **TAPE**	not applicable	**TAPE**, DWLS, Scaden
			Lung	not applicable	MuSiC, CIBERSORTx, Scaden	not applicable	MuSiC, DWLS, RNAsieve
			Marrow	not applicable	DWLS, Scaden, **TAPE**	not applicable	DWLS, **TAPE**, MuSiC
		similar distinguishment*	Marrow	not applicable	**TAPE**, DWLS, CIBERSORTx	not applicable	**TAPE**, CIBERSORTx, Scaden
		similar transferring*	Marrow	not applicable	DWLS, **TAPE**, Scaden	not applicable	DWLS, **TAPE**, Scaden
	counts2umi	normal	Limb_Muscle	**TAPE**, DWLS, Scaden	DWLS, **TAPE**, MuSiC	**TAPE**, Scaden, DWLS	**TAPE**, DWLS, Scaden
			Lung	MuSiC, **TAPE**, Scaden	MuSiC, DWLS, Bisque	MuSiC, **TAPE**, Scaden	MuSiC, **TAPE**, Scaden
			Marrow	MuSiC, **TAPE**, Scaden	MuSiC, **TAPE**, Scaden	**TAPE**, Scaden, MuSiC	**TAPE**, MuSiC, Scaden
		rare*	Limb_Muscle	not applicable	MuSiC, DWLS, **TAPE**	not applicable	MuSiC, **TAPE**, Scaden
			Lung	not applicable	DWLS, MuSiC, CIBERSORTx	not applicable	CIBERSORTx, DWLS, MuSiC
			Marrow	not applicable	MuSiC, CIBERSORTx, Scaden	not applicable	Scaden, DWLS, MuSiC
		similar distinguishment*	Marrow	not applicable	MuSiC, **TAPE**, CIBERSORTx	not applicable	**TAPE**, MuSiC, CIBERSORTx
		similar transferring*	Marrow	not applicable	MuSiC, **TAPE**, Scaden	not applicable	MuSiC, **TAPE**, Scaden

*only compute the average performance of all data points because of the small number of data points

Supplementary Table 9: Performance summary of TAPE and CIBESORTx on the DEG detection task. The performance is evaluated by the average AUROC in CD8 T cells. Since DEGs are only associated with different conditions which are not related to cell types' signature genes, we usually care about the case that DEGs are randomly selected.

Scenario	DEG type	Similar cell type	DEG number	Performance
1	random	no	100	TAPE > CIBERSORTx
			1000	TAPE > CIBERSORTx
			5000	TAPE ~ CIBERSORTx
2	signature	no	about 200	TAPE ~ CIBERSORTx
3	random	yes	100	both failed, TAPE > CIBERSORTx
			1000	both failed, TAPE > CIBERSORTx
4	signature	yes	about 150	CIBERSORTx > TAPE

Supplementary References

[1] Domínguez Conde, C., Xu, C., Jarvis, L. B., Rainbow, D. B., Wells, S. B., Gomes, T., ... & Teichmann, S. A. (2022). Cross-tissue immune cell analysis reveals tissue-specific features in humans. *Science*, *376*(6594), eabl5197.

[2] Love, M. I., Huber, W., & Anders, S. (2014). Moderated estimation of fold change and dispersion for RNA-seq data with DESeq2. *Genome biology*, *15*(12), 1-21.

[3] Zhao, B., Erwin, A., & Xue, B. (2018). How many differentially expressed genes: a perspective from the comparison of genotypic and phenotypic distances. *Genomics*, *110*(1), 67-73.
