# Supplementary material for: Deep autoencoder for interpretable tissue-adaptive deconvolution and cell-type-specific gene analysis
Source: Nat Commun. 2022 Nov 8;13:6735. doi: 10.1038/s41467-022-34550-9 (PMC9641692; doi:10.1038/s41467-022-34550-9)
Supplement: Supplementary file 3 — Reporting Summary [file 41467_2022_34550_MOESM3_ESM.pdf]

nature portfolio

Corresponding author(s): Ting-Fung Chan, Yu Li

Last updated by author(s): Oct 13, 2022

Reporting Summary

Nature Portfolio wishes to improve the reproducibility of the work that we publish. This form provides structure for consistency and transparency in reporting. For further information on Nature Portfolio policies, see our Editorial Policies and the Editorial Policy Checklist.

Statistics

For all statistical analyses, confirm that the following items are present in the figure legend, table legend, main text, or Methods section.

n/a	Confirmed	
☐	☒	The exact sample size (n) for each experimental group/condition, given as a discrete number and unit of measurement
☐	☒	A statement on whether measurements were taken from distinct samples or whether the same sample was measured repeatedly
☐	☒	The statistical test(s) used AND whether they are one- or two-sided<br>*Only common tests should be described solely by name; describe more complex techniques in the Methods section.*
☒	☐	A description of all covariates tested
☐	☒	A description of any assumptions or corrections, such as tests of normality and adjustment for multiple comparisons
☒	☐	A full description of the statistical parameters including central tendency (e.g. means) or other basic estimates (e.g. regression coefficient) AND variation (e.g. standard deviation) or associated estimates of uncertainty (e.g. confidence intervals)
☐	☒	For null hypothesis testing, the test statistic (e.g. F, t, r) with confidence intervals, effect sizes, degrees of freedom and P value noted<br>*Give P values as exact values whenever suitable.*
☒	☐	For Bayesian analysis, information on the choice of priors and Markov chain Monte Carlo settings
☒	☐	For hierarchical and complex designs, identification of the appropriate level for tests and full reporting of outcomes
☒	☐	Estimates of effect sizes (e.g. Cohen's d, Pearson's r), indicating how they were calculated

Our web collection on statistics for biologists contains articles on many of the points above.

Software and code

Policy information about availability of computer code

Data collection	no software was used
Data analysis	numpy>=1.19, open source python package for numerical calculations. pytorch>=1.8.0, open source deep learning framework in python. pandas>=1.3.1, open source python package for data analysis. matplotlib>=3.4, open source python package for visualization. seaborn>=0.11, open source python package for visualization. anndata>=0.7.6, open source python package for storing sequencing data. tqdm>=4.6, open source python package for displaying processing bar. scikit-learn>=0.23, open source python package for machine learning. scTAPE==1.1.0, custom code (open source), is used to analyze the bulk RNA-seq data (https://github.com/poseidonchan/TAPE). Scaden==1.1.2, published and open source model for analyzing bulk RNA-seq data, we implemented it following its description (original: https://github.com/KevinMenden/scaden, ours: https://github.com/poseidonchan/TAPE). RNA-Sieve==0.1.4, open source python package or Wolfram Mathematica language (https://github.com/songlab-cal/rna-sieve), used to analyze bulk RNA-seq data. CIBERSORTx, a web based software (https://cibersortx.stanford.edu/) for bulk RNA-seq data analysis (version: 2022-8-20, time consumption was tested on 2021-10-5). DWLS (no specific version), an open source R package for bulk RNA-seq data analysis (https://github.com/dtsoucas/DWLS). MuSiC==1.0.0, an open source R package that utilizes cross-subject scRNA-seq to estimate cell type proportions in bulk RNA-seq data. (https://github.com/xuranw/MuSiC). Bisque (no specific version), published and open source R package for estimation of cell composition from bulk expression data with single-cell information (https://github.com/cozygene/bisque). Single sample gene sets enrichment analysis 2.0 (ssGSEA2.0), open source R package (github: https://github.com/broadinstitute/ssGSEA2.0). DEseq2==1.36.0, open source analysis package for differentially expressed gene

For manuscripts utilizing custom algorithms or software that are central to the research but not yet described in published literature, software must be made available to editors and reviewers. We strongly encourage code deposition in a community repository (e.g. GitHub). See the Nature Portfolio guidelines for submitting code & software for further information.

Data

Policy information about availability of data

All manuscripts must include a data availability statement. This statement should provide the following information, where applicable:

- Accession codes, unique identifiers, or web links for publicly available datasets
- A description of any restrictions on data availability
- For clinical datasets or third party data, please ensure that the statement adheres to our policy

All the datasets we used are described in the Method part. Only the ROSMAP human brain dataset is not public, researchers need to download it from Synapse (ID: syn3219045) with a request. For convenience, we listed these datasets on the webpage: https://sctape.readthedocs.io/datasets/.

Field-specific reporting

Please select the one below that is the best fit for your research. If you are not sure, read the appropriate sections before making your selection.

☒ Life sciences ☐ Behavioural & social sciences ☐ Ecological, evolutionary & environmental sciences

For a reference copy of the document with all sections, see nature.com/documents/nr-reporting-summary-flat.pdf

Life sciences study design

All studies must disclose on these points even when the disclosure is negative.

Sample size	No data collection was involved in the present study. No statistical methods was used to predetermine the sample size or the number of datasets. Sample size was determined by previous published datasets. Datasets were chosen to show the functionality of our method.
Data exclusions	All samples from the chosen datasets were used, no exclusion was done prior to analysis.
Replication	No replication of tested data was performed. We did not collect experimental data, replication was determined by previously published datasets.
Randomization	Randomization of samples is not applicable to our study since we did not collect experimental data.
Blinding	Condition groups were determined by previously published datasets, we did not modify the group information.

Reporting for specific materials, systems and methods

We require information from authors about some types of materials, experimental systems and methods used in many studies. Here, indicate whether each material, system or method listed is relevant to your study. If you are not sure if a list item applies to your research, read the appropriate section before selecting a response.

Materials & experimental systems

n/a	Involved in the study	
☒	☐	Antibodies
☒	☐	Eukaryotic cell lines
☒	☐	Palaeontology and archaeology
☒	☐	Animals and other organisms
☒	☐	Human research participants
☒	☐	Clinical data
☒	☐	Dual use research of concern

Methods

n/a	Involved in the study	
☒	☐	ChIP-seq
☒	☐	Flow cytometry
☒	☐	MRI-based neuroimaging
